# Dr. C. H. Parry on the Powers of the Arteries, in Answer to Mr. Kerr

**Published:** 1816-09

**Authors:** 


THE LONDON

### Medical and Physical Journal.

3 OF VOL. XXXVI.] SEPTEMBER, 1816. [NO. 211.

"For many fortunate discoveries in medicine, and for the detection of nume-
"rous errors, the world is indebted to the rapid circulation of Monthly
"Journals; and there never existed any work to which the Faculty in
"EUROPE and AMERICA were under deeper obligations than to the
"Medical and Physical Journal of London, now forming a long, but an
"invaluable, series."—RUSH.

*For the London Medical and Physical Journal.*

### Dr. C. H. PARRY *on the Powers of the Arteries, in Answer to Mr. Kerr.*

IF your correspondent, Mr. Kerr, will re-peruse Mr. Astley Cooper's paper, vol. ii. p. 249, of the Medico-Chirurgical Transactions, I believe he will find himself in an error respecting the effects of the ligature of the abdominal aorta in a dog, there specified. A certain portion of the artery appeared to have been obliterated; but the circulation was carried on beyond that part by the enlargement of certain arterial branches, which were given off before it, and which united, by anastomosis, with other branches beyond. I do not, however, see any thing which proves either the production of a new artery, or the return of any branches of the old artery into the more distant portion of the same trunk.

The great question, however, remains as Mr. Kerr states it: Why does the animal not only survive the operation, but bear it with so little apparent distress?

We know that it was performed in the abdomen, but we are not told at what precise spot, and, therefore, are ignorant what arterial branches the aorta had previously distributed, and by what anastomoses the blood might enter the inferior cava, so as afterwards to circulate through the other routes, common to the healthy state. Neither are we told whether the pulse was quickened by the operation. This point is of great consequence; for a quantity of blood, as one, emitted from the left ventricle twice in a given time, would be just equal to two, emitted once in the same time.

It is also certain that arteries very readily accommodate themselves to the quantity of blood which is required to circulate through them; for they not only immediately contract to a certain extent, when the quantity is lessened, but very quickly expand, when the quantity is increased. The truth of the former of these positions appears from experiments xxiv. and xxvii.; and of the second from experiments xxiii. and xxiv., in the work on Arteries to which Mr. Kerr alludes.

The ligature of the abdominal aorta had been practised by Haller, and, as that candid author himself acknowledges, long before his time by Heno; both of whom observed, from the operation, an approach to a paralysis of the lower extremities, but little other inconvenience.

It was, however, far otherwise with the thoracic aorta, the ligature of which, in a cat, caused the animal to fall into a stupor, and general insensibility. (Haller, sur le mouvement du sang arteriel. Edit. Lausanne, p. 45, 46.)

The proposition of Mr. Kerr to examine the state of the carotid artery at different periods after such an operation, in order to see at what time this important process of regeneration commences, is a very interesting one. My own views have chiefly gone to what may be considered as the converse of that experiment; and have led me rather to observe what may occur in the course of a longer trial of the natural process. I have, therefore, now, one ram, of which one carotid only was tied in April 1815; another, both carotids of which were tied in September 1815; and a third, under the same process, begun one month later.

The chief view of these long trials is to observe whether a complete regeneration of the artery may not ultimately take place, so as to render unnecessary the accessory branches, which have, in the interim, served as substitutes.

*Bath; August* 7, 1816.

---

*For the London Medical and Physical Journal.*

